# The CIDR-GPG Protocol Improves Reproductive Efficiency in Yaks and Lowers the Body Condition Requirements for Success

**DOI:** 10.3390/ani16111582

**Published:** 2026-05-22

**Authors:** Zhuoyuan Song, Jiarui Cheng, Chuanlong Wang, Qiguo Yin, Zhimin Chen, Rui Li, Yaxin Yang, Yawen Ji, Jiakui Li, Yun Liu, Gongjue Caiwang, Yun Gao, Guohua Hua

**Affiliations:** 1 Key Laboratory of Agriculture Animal Genetics, Breeding and Reproduction, College of Animal Science and Technology, Huazhong Agriculture University, Ministry of Education, Wuhan 430070, China; zysong@webmail.hzau.edu.cn (Z.S.); chengjiarui@webmail.hzau.edu.cn (J.C.); clwang@webmail.hzau.edu.cn (C.W.); 2023job@webmail.hzau.edu.cn (Q.Y.); chenzhimin0609@webmail.hzau.edu.cn (Z.C.); 2023302220521@webmail.hzau.edu.cn (R.L.); yangyaxin@webmail.hzau.edu.cn (Y.Y.); 13048777638@webmail.hzau.edu.cn (Y.J.); liuyun@mail.hzau.edu.cn (Y.L.); 2 National Center for International Research on Animal Genetics, Breeding and Reproduction, Wuhan 430070, China; 3 Hubei Hongshan Laboratory, Huazhong Agricultural University, Wuhan 430070, China; 4 Frontiers Science Center for Animal Breeding and Sustainable Production, Huazhong Agricultural University, Wuhan 430070, China; 5 Dangxiong County Yak Frozen Semen Station, Lhasa 850000, China

**Keywords:** yak, estrus synchronization, pregnancy rate, body condition, chest girth

## Abstract

Yaks are a critical livestock species for plateau regions; however, their poor reproductive efficiency—characterized by low estrus and pregnancy rates—significantly hampers genetic improvement and industry sustainability. Although synchronization protocols are widely utilized in cows to enhance reproductive performance, research on yaks is limited. Our study systematically evaluated 3 estrus synchronization protocols (CIDR-GPPG, CIDR-GPG, GPPG) on 99 female yaks. The GPPG treatment resulted in the lowest estrus detection (40.63%) and pregnancy rate (15.38%), while the CIDR-GPG protocol significantly improved these rates, achieving an estrus rate of 65.71% and a pregnancy rate of 52.17%. Notably, serum estradiol concentrations were significantly elevated in the CIDR-GPG group compared to the GPPG group. Given the importance of body condition for synchronization outcomes, we analyzed correlation between body measurements and reproductive performance. Among 10 body measurements, chest girth demonstrated the strongest positive correlation with both estrus (R = 0.75) and pregnancy success (R = 0.72). Further logistic regression modeling based on chest girth generated robust predictors for estrus (AUC = 0.934) and pregnancy (AUC = 0.923). Importantly, CIDR-GPG protocol effectively lowered the body condition threshold required for successful reproductive outcomes, indicating that yaks with smaller chest girths can still achieve comparable probabilities of estrus and pregnancy when employing this protocol. Overall, our findings supported the CIDR-GPG protocol as an effective strategy for optimizing yak reproduction and highlighted chest girth as a valuable indicator for predicting reproductive outcomes, providing a practical approach to enhance reproductive management, even in animals with suboptimal body conditions.

## Simple Summary

Yaks are essential livestock for plateau communities, but low reproductive efficiency limits their productivity and farmers’ income. This study compared 3 estrus synchronization protocols and identified CIDR-GPG as the most effective for improving estrus and pregnancy rates. Additionally, we found that body condition significantly influenced the reproductive performance, with chest girth identified as the most relevant indicator. Importantly, our findings demonstrated that CIDR-GPG reduces the chest girth threshold required for successful reproduction, allowing yaks with relatively poor body condition to achieve comparable reproductive outcomes. This work provides an effective strategy to enhance reproductive efficiency and sustainability for plateau yak industry.

## 1. Introduction

*Domestic yaks* (Bos grunniens) are primarily found in the plateau regions of Asia and have spread to 14 surrounding countries, including China, Pakistan, and Nepal. As one of the few livestock species capable of thriving in extreme environments with altitudes ranging from 3000 to 5400 m, yaks are vital for the survival of over 65 million inhabitants of these high-altitude areas [1,2]. In China alone, the yak population is approximately 14 million, constituting over 90% of the global total [3]. Their unique physiological adaptations, such as cold tolerance and efficient roughage utilization, enable yaks to play a crucial role in the nutrient cycle of alpine ecosystems. By grazing and excreting, they contribute to maintaining vegetation diversity and serve as a key species in supporting the food webs of plateau carnivores [4,5].

Despite their ecological importance, the yak industry faces significant challenges due to low reproductive efficiency. Yaks exhibit distinct seasonal breeding patterns, with a breeding season limited to June through November, resulting in a short and concentrated breeding window [6,7]. Moreover, yaks reach sexual maturity much later than cattle, with heifers typically not mating until they are 3–4 years old [8,9]. Additionally, low estrus detection and pregnancy rates contribute to prolonged calving intervals, further diminishing breeding efficiency and profitability.

Estrus synchronization-timed artificial insemination (TAI) technology has been well-established in ruminants such as cattle and buffalo and has been shown to improve reproductive efficiency [10,11,12]. However, its application in yaks remains relatively unexplored, with limited studies and inconsistent reports on protocol efficacy [13,14]. This inconsistency may stem from unaccounted-for variability in individual body condition, a factor known to significantly influence reproductive outcomes in other species. In cows, body measurement indicators (such as body height, body length, chest girth, etc.) are commonly used to estimate body size and body weight, and several studies have reported significant correlations between body measurements, body weight, and body condition score (BCS) [15,16]. A “safety threshold” of BCS ≥ 2.5 on the day of AI is recommended for dairy cows to achieve higher pregnancy rates [17]. Chest girth has been reported to be positively correlated with body weight and body condition score (BCS) in cattle and may serve as an indirect indicator of nutritional and reproductive status [18]. While perinatal nutritional supplementation has been shown to significantly improve metabolic status and increase pregnancy rates in yaks [19], the effects of body condition on reproductive and synchronization outcomes remain unclear.

Therefore, this study was conducted to evaluate 3 estrus synchronization protocols (CIDR-GPPG, CIDR-GPG, and GPPG) in yaks to identify the optimal protocol during the reproductive season, and to investigate the correlations between a comprehensive set of body measurements and synchronization success (estrus and pregnancy). The purpose was to develop predictive models based on the most relevant body measurements, which would provide a practical tool for formulating effective reproductive management strategies to enhance successful reproduction in yaks.

## 2. Materials and Methods

This study was approved by the Animal Experimental Ethical Inspection Committee of the Laboratory Animal Centre at Huazhong Agricultural University (HZAUMO-2023-0303) and conducted following its Laboratory Animal Use Guidelines.

### 2.1. Experimental Animals and Estrus Synchronization Protocols

**Figure 1:**
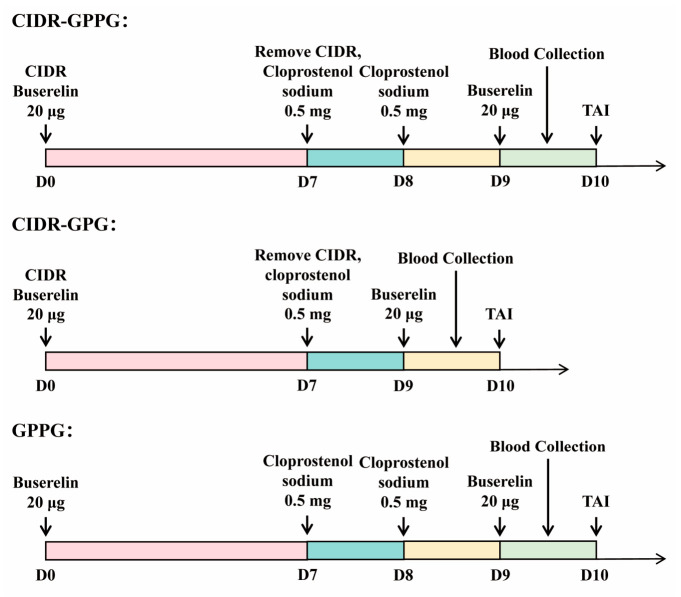
Experimental design of the 3 estrus synchronization–timed artificial insemination (TAI) protocols (CIDR-GPPG, CIDR-GPG, and GPPG) applied in female yaks. PG: Cloprostengol sodium, Buserelin: a GnRH analog, CIDR: controlled internal drug release device containing 1.38 g progesterone.

The experiment was conducted from August to November 2024 at a yak breeding base in Dangxiong County, Lhasa City, Xizang Autonomous Region, located at an altitude of 4300 m, with an average seasonal temperature ranging from 6 °C to 17 °C. Ninety-nine healthy, non-pregnant multiparous female yaks (aged 4–5 years) with no history of ovarian or reproductive tract disorders and normal prior reproductive performance were selected. The yaks were randomly assigned to one of 3 estrus synchronization protocols: CIDR-GPPG, CIDR-GPG, or GPPG (Figure 1). The detailed procedures for each protocol are outlined below.

Ninety-nine healthy, non-pregnant multiparous female yaks (aged 4–5 years), all with a previous calving history and normal reproductive performance, were selected for this study.

CIDR-GPPG: On Day 0 at 09:00, a controlled internal drug release device (CIDR) which containing 1.38 g progesterone (Sansheng Biological Technology Co., Ltd., Ningbo, China) was inserted intravaginal, and 20 μg of Buserelin (a GnRH analog; Sansheng Biological Technology Co., Ltd., Ningbo, China) was administered intramuscularly. On Day 7 at 09:00, the CIDR was removed, and 0.5 mg (5 mL) of cloprostenol sodium (a synthetic PGF_2_α analogue, Sansheng Biological Technology Co., Ltd., Ningbo, China) was administered intramuscularly. On Day 8 at 09:00, a second intramuscular injection of 0.5 mg cloprostenol sodium was administered. On Day 9 at 17:00, yaks received an intramuscular injection of 20 μg Buserelin. On Day 10, two AI were performed at 09:00 and 17:00, respectively.

CIDR-GPG: Females were treated similarly to the CIDR-GPPG protocol, with the exception of cloprostenol sodium on Day 8. Briefly, the protocol involved an initial injection of Buserelin on Day 0, along with the insertion of a CIDR. Seven days later, cloprostenol sodium was administered at the time of CIDR removal. A second injection of Buserelin was given on Day 9, followed by two artificial inseminations on Day 10.

GPPG: On Day 0 at 09:00, yaks received an intramuscular injection of 20 μg Buserelin (Sansheng Biological Technology Co., Ltd., Ningbo, China). On Day 7 at 09:00, 0.5 mg cloprostenol sodium was administered intramuscularly, followed by a second identical injection on Day 8 at 09:00. On Day 9 at 17:00, an intramuscular injection of 20 μg Buserelin was administered. On Day 10 at 09:00 and 17:00, two fixed-time AI were performed at 09:00 and 17:00.

All yaks were housed in a standard free-stall system with ad libitum access to water and total mixed ration (TMR; detailed feed composition and nutrient levels are provided in Supplementary Table S1). The animals were maintained under the same management and feeding conditions throughout the experimental period. Free-stall pens were bedded with dry straw and cleaned regularly.

### 2.2. Estrus Identification and Artificial Insemination

Blood samples were collected from the jugular vein before artificial insemination to determine circulating estradiol (E_2_) concentrations. After collection, blood samples were centrifuged at 3000× *g* for 15 min, and the serum was separated and stored at −20 °C until analysis. Serum E_2_ concentrations were measured using a commercial enzyme-linked immunosorbent assay (ELISA) kit according to the manufacturer’s instructions. All serum samples were analyzed in the same assay batch to avoid inter-assay variation. Estrus was identified based on behavioral observations and vaginal examination [20]. Following the final hormone injection, continuous monitoring was performed to detect increased activity, mounting behavior, and vulvar swelling with mucous discharge. Vaginal examination was conducted on yaks exhibiting or suspected of exhibiting estrus signs. Estrus was confirmed by observing a congested and swollen vaginal mucosa, an open and congested cervix, and the presence of mucus. Only females confirmed in estrus were inseminated. For these yaks, sperm motility was assessed prior to artificial insemination (Table S2). Artificial insemination was performed twice at 8 h intervals. Frozen semen straws were randomly allocated and evenly distributed among the different treatment groups. All inseminations were performed by the same experienced technician.

### 2.3. Body Measurement of Experimental Animals

Ten body conformation traits of female yaks were measured following standardized zootechnical procedures, as previously described in cattle morphometric studies [21]. Body height was defined as the vertical distance from the withers (the highest point of the shoulder) to the ground. Body straight length was measured as the horizontal distance from the point of the shoulder to the posterior edge of the ischial tuberosity along a straight line. Body slope length referred to the distance from the point of the shoulder to the posterior edge of the ischial tuberosity following the slope of the body. Head length was measured as the straight-line distance from the top of the head to the tip of the nose. Sacral height was defined as the vertical distance from the highest point of the sacrum to the ground. Cannon girth was measured as the circumference of the narrowest part of the cannon bone (metacarpus). Chest girth was measured as the body circumference immediately behind the forelimbs at the widest part of the chest. Abdominal girth was measured as the circumference of the abdomen immediately behind the last rib at the widest part of the abdomen. Hip width was measured as the distance between the outer edges of the ischial tuberosi ties, and pelvic width was defined as the distance between the outer edges of the hip joints.

### 2.4. Prediction Model Construction

Pearson correlation analysis was first performed to evaluate the associations between 10 body measurements of yaks and their estrus or pregnancy status. Based on the correlation coefficients, the most relevant body measurement was identified, and a correlation heatmap was generated to visualize these relationships. Subsequently, a logistic regression model was constructed to predict estrus or pregnancy outcomes.

Estrus status or pregnancy status was encoded as binary labels (Not Estrus = 0, Estrus = 1 or Not Pregnant = 0, Pregnant = 1). After data preprocessing, chest girth was selected as the input feature, while estrus probability or pregnancy probability served as the output variable. Logistic regression analysis was conducted using R software (version 4.4.3). The dataset was randomly divided into training and test sets at a ratio of 8:2, with stratification applied to preserve the distribution of outcome variables. The logistic regression model was used to characterize the relationship between chest girth (*x*, in cm) and the probability (P) of estrus or pregnancy. This relationship was modeled using the Sigmoid function, allowing the estimation of outcome probabilities ranging from 0 to 1. The basic form of the logistic regression model was expressed as:

$$p=1/(1+{exp}^{-}(\theta 0\;+\;\theta 1\;x))$$

where θ0 is the intercept, and θ1 is the regression coefficient corresponding to chest girth. Model parameters were estimated using the maximum likelihood estimation method.

### 2.5. Prediction Model Evaluation

After model construction using the training set, independent validation was performed on the test dataset. Model performance was evaluated in terms of discriminatory ability and classification performance.

Discriminatory power was assessed by plotting the receiver operating characteristic (ROC) curve and calculating the area under the curve (AUC). The AUC value ranges from 0.5 to 1.0, where 0.7–0.8 indicates the model has good discriminatory ability, and 0.8–0.9 indicates excellent discriminatory ability.

Additionally, classification performance was further evaluated using accuracy, precision, recall, and the F1 score. Accuracy was defined as the proportion of correctly predicted samples among all samples. Precision was defined as the proportion of true positive samples among all samples predicted as positive. Recall was defined as the proportion of true positive samples correctly identified by the model among all actual positive samples. The F1 score, calculated as the weighted harmonic mean of precision and recall, was used to provide a comprehensive assessment of classification performance.

### 2.6. Statistical Analysis

Logistic regression analysis was conducted using R software (version 4.4.3, Vienna, Austria). Other statistical analyses were performed using GraphPad Prism 5 software (GraphPad Software, San Diego, CA, USA). Continuous variables, including serum estradiol concentration and the 10 body measurement traits (body height, body straight length, body slope length, head length, sacral height, cannon girth, chest girth, abdominal girth, hip width, and pelvic width), were analyzed using the unpaired two-tailed Student’s t-test or two-way ANOVA, while categorical variables were compared using the chi-square (χ^2^) test.

A post hoc power analysis was conducted using G*Power software (version 3.1.9.7; Heinrich-Heine-Universität Düsseldorf, Düsseldorf, Germany), based on the observed differences in estrus and pregnancy rates between groups using a two-proportion Z-test. In addition, cross-validation was performed in R (version 4.4.3; Vienna, Austria) using stratified k-fold procedures (k = 5 or 3 depending on sample size).

## 3. Results

### 3.1. Comparison of Different Synchronization Protocols on Yak Estrus

No significant differences in body measurement traits were observed among the groups before treatment (Table 1). Overall, 52 of 99 yaks (52.53%) exhibited estrus. The estrus rate was highest in the CIDR-GPG group (65.71%, 23/35), intermediate in the CIDR-GPPG group (50.00%, 16/32), and lowest in the GPPG group (40.63%, 13/32). A significant difference was detected between the CIDR-GPG and GPPG groups (χ^2^ test, *p* = 0.04). This indicates that the CIDR-GPG protocol may have a marked positive effect on inducing estrus in yaks (Table 2).

### 3.2. Comparison of Circulation Estradiol Levels in Different Synchronization Protocols

**Figure 2:**
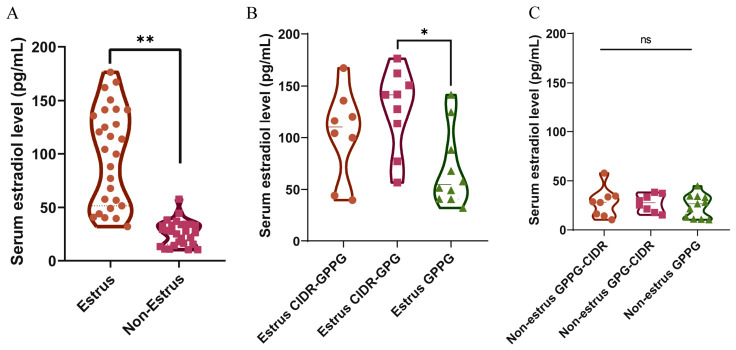
Serum E_2_ concentrations in female yaks under 3 estrus synchronization protocols. (**A**), Comparison of serum E_2_ levels between estrus and non-estrus yaks. (**B**), Comparison of serum E_2_ levels in estrus yaks among the 3 estrus synchronization protocols. (**C**), Comparison of serum E_2_ levels in non-estrus yaks among the 3 estrus synchronization protocols. Data are presented as mean ± SEM. “ns” indicates no significant difference (*p* > 0.05), whereas “*” and “**” indicate significant differences at *p* < 0.05 and *p* < 0.01, respectively.

ELISA results showed that E_2_ levels in estrus yaks were significantly higher than those in non-estrus yaks (Figure 2A). E_2_ levels in estrus yak under the CIDR-GPG protocol were significantly higher compared to those in the GPPG protocol (Figure 2B), suggesting enhanced follicular development induced by this protocol. There were no significant differences in average E_2_ levels among the non-estrus yaks across the 3 groups (Figure 2C).

### 3.3. Comparison of Different Synchronization Protocols on Yak Pregnancy

Of the 52 yaks inseminated, 20 became pregnant, resulting in an overall pregnancy rate of 38.5%. Statistical results showed that 20 yaks became pregnant, yielding an overall pregnancy rate of 38.46% (Table 3). Among these protocols, the CIDR-GPG protocol achieved the highest pregnancy rate at 55.56% (12/23), followed by the CIDR-GPPG protocol at 35.29% (6/16) and the GPPG protocol at 23.53% (2/13). A significant difference was found between CIDR-GPG and GPPG protocol (*p* = 0.03), suggesting that CIDR-GPG protocol may have potential advantages in promoting pregnancy in yak (Table 3).

### 3.4. Correlation Analysis Between Yak Body Measurements and Estrus Status of Synchronization

To systematically evaluate how individual phenotypic characteristics, influence the synchronization of estrus in yaks, we investigated 10 body measurement indicators in 99 female yaks, including body height, sacral height, body straight length, body slope length, chest girth, abdominal girth, cannon girth, head length, hip width, and pelvic width. These indicators were compared between the estrus group (*n* = 52) and the non-estrus group (*n* = 47). Eight of the 10 body measurements were significantly greater in yaks that exhibited estrus compared to those that did not (*p* < 0.05), including body height, body straight length, body Slope length, Sacral height, cannon girth, chest girth, abdominal girth, and pelvic width (Table 4).

**Figure 3:**
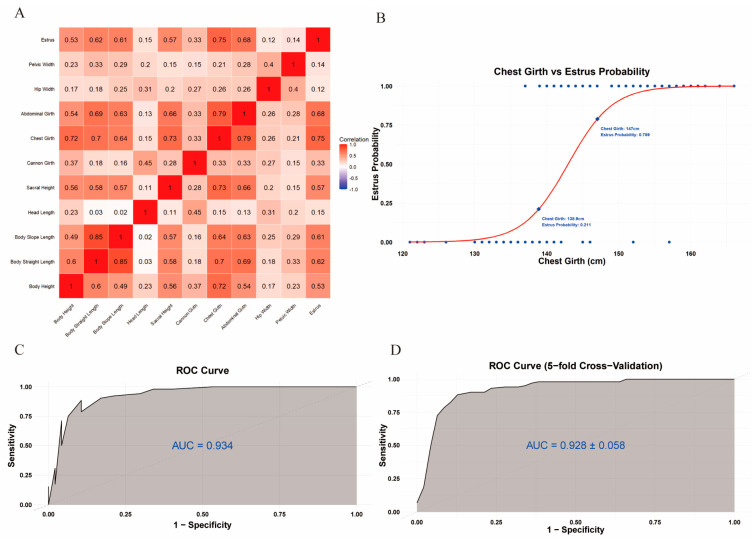
Chest girth–based logistic regression model and screening thresholds for estrus prediction in female yaks (*n* = 99). (**A**), Correlation heat map illustrating the relationships between 10 body measurements and estrus status. (**B**), Logistic regression curve showing the association between chest girth and estrus probability, with the upper and lower points of maximum curvature indicated. (**C**), Receiver operating characteristic (ROC) curve evaluating the discriminatory performance of the estrus prediction model. (**D**), ROC curves for overall estrus prediction based on stratified five-fold cross-validation. The AUC values are presented as mean ± SD. Upper Point of Maximum Curvature: In the logistic regression model, the point at which the upper segment of the sigmoid curve exhibits the greatest curvature. Lower Point of Maximum Curvature: In the logistic regression model, the point at which the lower segment of the sigmoid curve exhibits the greatest curvature.

Correlation analysis revealed associations between body measurement traits and estrus status (Figure 3A). Strong positive correlations with estrus were observed for chest girth (r = 0.75), abdominal girth (r = 0.68), body straight length (r = 0.62), and body slope length (r = 0.61). Moderate correlations were found for sacral height (r = 0.57) and body height (r = 0.53), while cannon girth (r = 0.33) showed a weak correlation. Head length (r = 0.15), pelvic width (r = 0.14), and hip width (r = 0.12) exhibited negligible effects on estrus.

**Figure 4:**
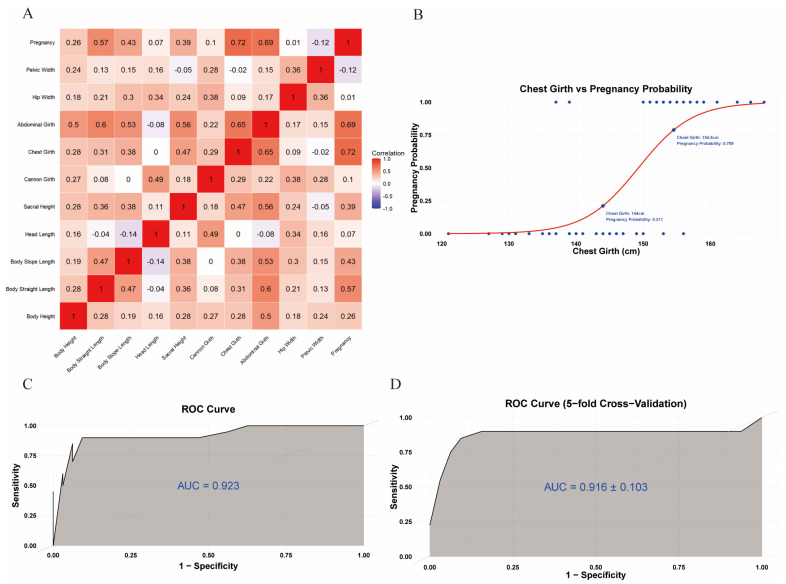
Chest girth–based logistic regression model and screening thresholds for pregnancy prediction in female yaks (*n* = 52). (**A**), Correlation heatmap illustrating the relationships between 10 body measurements and pregnancy status. (**B**), Logistic regression curve showing the association between chest girth and pregnancy probability, with the upper and lower points of maximum curvature indicated. (**C**), Receiver operating characteristic (ROC) curve evaluating the discriminatory performance of the pregnancy prediction model. (**D**), ROC curves for overall pregnancy prediction based on stratified five-fold cross-validation. The AUC values are presented as mean ± SD. Upper Point of Maximum Curvature: In the logistic regression model, the point at which the upper segment of the sigmoid curve exhibits the greatest curvature. Lower Point of Maximum Curvature: In the logistic regression model, the point at which the lower segment of the sigmoid curve exhibits the greatest curvature.

Given that chest girth demonstrated the strongest correlation with estrus, we established a binary logistic regression model based on chest girth and estrus status. The result showed that the probability of estrus increased with the chest girth, aligning well with the observed phenotypic correlations. Within the chest girth range of 135 cm to 150 cm, the probability of estrus rises sharply, suggesting a threshold effect in this range (Figure 4B). The fitting equation for this model was as follows:

$$p=1/(1+{exp}^{-}(-46.22+0.32x))$$

where p indicated the possibility of estrus.

The model showed strong discriminatory performance, with an AUC of 0.934 (Figure 3C). The classification model also achieved high predictive performance, with Accuracy, Precision, Recall, and F1 scores of 0.889, 0.902, 0.885, and 0.893, respectively (Table 5).

Curvature analysis of the logistic regression model identified threshold values of chest girth associated with high and low estrus probabilities. The results showed that the upper point of maximum curvature corresponded to a chest girth of 147.0 cm, where the estrus probability was 78.9% (Figure 3B), which was close to the average chest girth of estrus yaks (150.5 ± 6.6 cm). Conversely, the lower point of maximum curvature corresponded to a chest girth of 138.9 cm, where the estrus probability was 21.1% (Figure 3B), indicating a 78.9% probability of not being in estrus.

To assess the robustness of the model, a five-fold cross-validation was performed. The mean AUC for overall estrus prediction was 0.92 ± 0.067 (Figure 3D).

### 3.5. Correlation Analysis Between Yak Body Measurements and Pregnancy Status of Synchronization

Consistent with their effects on estrus, all body measurements of pregnant yaks were significantly larger than those of non-pregnant yaks. Specifically, body straight length, body slope length, sacral height, chest girth, and abdominal girth were markedly greater in pregnant yaks compared to their non-pregnant counterparts (Table 6).

The correlation heatmap analysis further underscored that chest girth (r = 0.72) and abdominal girth (r = 0.69) were the strongest body measurement indicators for predicting pregnancy success. In comparison, other measurements such as head length, cannon girth, hip width, and pelvic width exhibited negligible effects on pregnancy outcomes (Figure 4A). Notably, chest girth displayed the strongest correlation with both estrus and pregnancy status, and it also demonstrated a significant synergistic relationship with abdominal girth. This suggests that together, these measurements reflect both torso girth and energy reserves, making them critical factors influencing estrus and pregnancy rates.

To quantify the relationship between chest girth and pregnancy status, we developed a binary logistic regression model utilizing data from inseminated yaks (n = 52). The results showed that probability of pregnancy increased with larger chest girth, and the model fitting followed a pattern consistent with observed phenotypic associations (Figure 4B). The fitted equation was:

$$p=1/(1+{exp}^{-}(-37.32+0.25x))$$

where p indicated the possibility of pregnancy.

Its AUC value of this model was 0.923 (Figure 4C), with all performance evaluation metrics indicating strong classification performance (Table 7). Furthermore, curvature analysis revealed that the upper point of maximum curvature corresponded to a chest girth of 154.5 cm, corresponding to a pregnancy probability of 78.9%. This measurement was consistent with the average chest girth of pregnant yaks, noted as 155.4 ± 8.0 cm. Conversely, the lower point of maximum curvature was found at a chest girth of 144.0 cm, indicating a pregnancy probability of 21.1% (Figure 4B).

The predictive performance of the model for pregnancy outcome was evaluated using stratified five-fold cross-validation. The mean AUC was 0.916 ± 0.103 (Figure 4D).

### 3.6. Correlation Analysis Between Body Measurements and Estrus/Pregnancy Status in Yaks Under the CIDR-GPG Protocol

**Figure 5:**
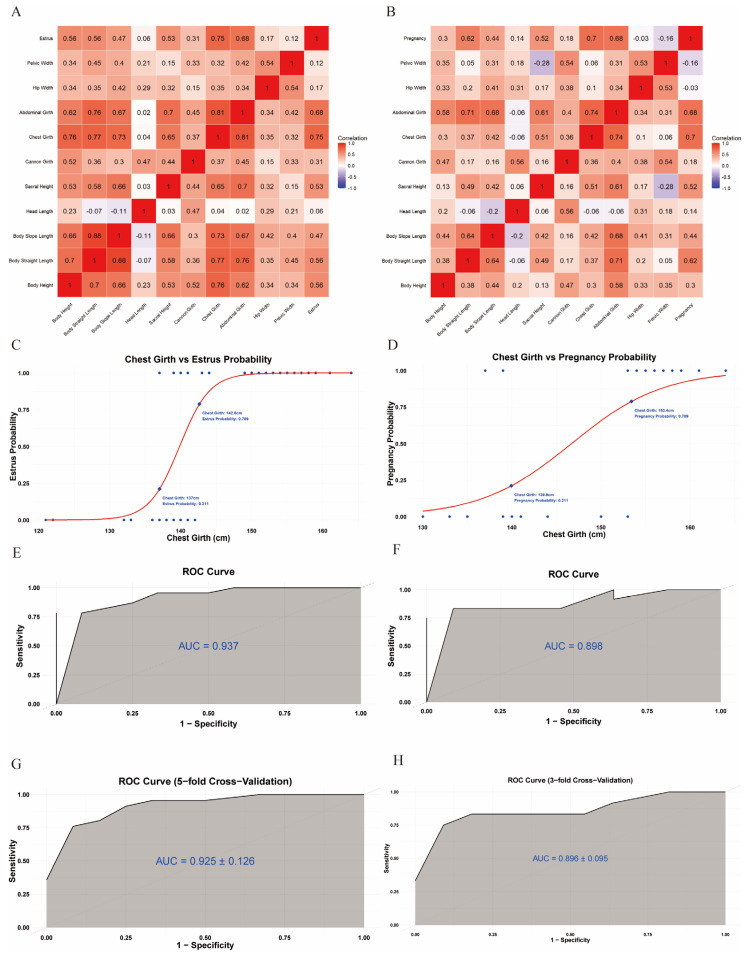
Chest girth–based logistic regression models and screening thresholds for estrus and pregnancy prediction in female yaks under the CIDR-GPG protocol (*n* = 35). (**A**), Correlation heatmap illustrating the relationships between 10 body measurements and estrus status under the CIDR-GPG protocol. (**B**), Correlation heatmap illustrating the relationships between 10 body measurements and pregnancy status under the CIDR-GPG protocol. (**C**), Logistic regression curve showing the association between chest girth and estrus probability, with the upper and lower points of maximum curvature indicated. (**D**), Logistic regression curve showing the association between chest girth and pregnancy probability, with the upper and lower points of maximum curvature indicated. (**E**), ROC curve evaluating the discriminatory performance of the estrus prediction model under the CIDR-GPG protocol. (**F**), ROC curve evaluating the discriminatory performance of the pregnancy prediction model under the CIDR-GPG protocol. (**G**), ROC curves for overall estrus prediction based on the CIDR-GPG synchronization protocol using stratified five-fold cross-validation. The AUC values are presented as 0.925 ± 0.126. (**H**), ROC curves for overall pregnancy prediction based on the CIDR-GPG synchronization protocol using stratified three-fold cross-validation. The AUC values are presented as 0.896 ± 0.095. Upper Point of Maximum Curvature: In the logistic regression model, the point at which the upper segment of the sigmoid curve exhibits the greatest curvature. Lower Point of Maximum Curvature: In the logistic regression model, the point at which the lower segment of the sigmoid curve exhibits the greatest curvature.

Under the CIDR-GPG protocol, chest girth emerged as the strongest predictor for both estrus (r = 0.75) and pregnancy (r = 0.70) (Figure 5A,B). Logistic regression models based on chest girth demonstrated high predictive performance, with AUC values of 0.937 for estrus and 0.898 for pregnancy (Figure 5E,F). Consistently, the overall classification performance of both models was favorable (Table 8).

The fitted logistic regression equation describing the relationship between chest girth and estrus probability was as follows:

$$p=1/(1+{exp}^{-}(-65.63+0.47x))$$

where p indicated the possibility of estrus.

The fitted logistic regression equation for predicting pregnancy probability based on chest girth was:

$$p=1/(1+{exp}^{-}(-28.95+0.20x))$$

where p indicated the possibility of pregnancy.

Notably, curvature analysis revealed that, in the estrus prediction model under the CIDR-GPG protocol, the upper and lower points of maximum curvature were 142.6 cm and 137.0 cm, respectively, both lower than the corresponding values in the overall population (147.0 cm and 138.9 cm). Similarly, in the pregnancy prediction model, the upper and lower curvature points were 153.4 cm and 139.9 cm, also below the total population averages of 154.5 cm and 144.0 cm, respectively. These results indicated that, under the CIDR-GPG protocol, yaks with smaller chest girths were still able to achieve comparable probabilities of estrus and pregnancy (Figure 5C,D).

To further validate model robustness, stratified five-fold cross-validation was performed for estrus prediction. The mean AUC values were 0.925 ± 0.126. For pregnancy prediction, stratified three-fold cross-validation was applied, and the model achieved a mean AUC of 0.896 ± 0.095 (Figure 5G,H).

## 4. Discussion

Despite its importance on plateau livestock industry, the reproductive management of yak is relatively primitive compared to cow. The low reproductive efficiency of yaks severely hampers the genetic improvement and breeding profitability. This study identified CIDR-GPG as the most effective synchronization method for plateau yaks among the 3 synchronization protocols evaluated. Furthermore, chest girth was identified as a key predictor of reproductive success. Two logistic regression models were developed based on chest girth, both demonstrating high accuracy. Importantly, our findings demonstrated that the CIDR-GPG protocol can lower the chest girth threshold necessary for achieving estrus and pregnancy, thereby expanding the population of animals that can benefit from enhanced reproductive management.

The superior performance of CIDR-based (CIDR-GPG and CIDR-GPPG) protocols over the CIDR-free (GPPG) protocol aligns with established mechanisms in cattle. This improvement is primarily attributable to the sustained release of progesterone from CIDR, which mimics the luteal phase, suppresses premature GnRH and LH pulses, and promotes synchronization of follicular waves. Following CIDR removal, the rapid decline in progesterone levels, together with PGF_2_α-induced luteolysis, facilitates the synchronous growth of dominant follicles to an optimal ovulatory diameter (13–16 mm), thereby enhancing ovulation synchrony [22,23]. In addition, progesterone priming may further enhance follicular competence by improving granulosa cell function and steroidogenic capacity, thereby promoting estradiol (E_2_) production. Consistent with this, we observed higher circulating E_2_ levels in the CIDR-GPG group, indicating improved follicular maturity and endocrine responsiveness, which are closely associated with enhanced estrus expression and ovulatory synchronization. Moreover, progesterone pretreatment has been shown to improve oocyte quality and endometrial receptivity, creating favorable conditions for subsequent fertilization and implantation [24,25]. Consistent with these mechanisms, the use of PRID in lactating dairy cows increased pregnancy rates from 31.7% to 38.9% [26]. Furthermore, large-scale studies in beef cattle have reported that incorporating CIDR into a GnRH–PGF_2_α-based synchronization framework significantly improves both estrus expression within 60 h after device removal and pregnancy rates following TAI (65.2% vs. 30.8%) [27]. Our results extend these well-characterized benefits to yaks, confirming the transferability of CIDR-based synchronization technology to this species.

Among the two CIDR-based protocols, CIDR-GPG outperformed CIDR-GPPG in estrus rate and pregnancy rate. This difference may be related to the number of PGF_2_α injections. The 5-day Ovsynch protocol typically requires two PGF_2_α injections to ensure complete luteolysis because of the presence of younger corpora lutea with reduced PG sensitivity. In contrast, the 7-day protocol used in this study corresponds to a more mature luteal phase, which responds sufficiently to a single PGF_2_α injection [28,29,30]. Excessive or repeated PGF_2_α administration may also disrupt endocrine coordination and uterine function, including inducing excessive uterine contractions and impairing endometrial receptivity, potentially compromising early embryo development [31,32]. Furthermore, inter-individual variation in luteal regression timing may lead to asynchronous dominant follicle maturation and LH surge, thereby reducing ovulation synchrony and pregnancy success [33,34]. In contrast, the CIDR-GPG protocol, with a simplified hormonal regimen, may provide a more stable and coordinated endocrine environment, which likely contributes to the higher E_2_ levels, improved estrus expression, and increased pregnancy rates observed in this study. Previous studies have also indicated that simplified synchronization strategies with fewer hormonal interventions can improve reproductive efficiency while maintaining acceptable estrus synchronization rates [35].

Our findings reinforce the well-established link between body size and reproductive efficiency in ruminants. Individuals with superior body condition are generally considered to possess greater body fat reserves, which may influence the hypothalamic–pituitary–gonadal (HPG) axis through metabolic signals such as leptin, potentially contributing to increased E_2_ synthesis and improved follicular development and estrus expression [36]. Consistent with this mechanism, we observed significantly higher serum E_2_ concentrations in estrous yaks, particularly in the CIDR-GPG group, suggesting that favorable body condition may be associated with enhanced endocrine responsiveness to estrus synchronization protocols. In contrast, when body condition deteriorates or animals experience negative energy balance (NEB), circulating levels of non-esterified fatty acids (NEFA) and β-hydroxybutyrate (BHBA) increase. These metabolic alterations suppress HPG axis activity and reduce the secretion of key hormones, including insulin-like growth factor 1 (IGF-1) and thyroxine (T_4_), ultimately being linked to impairing follicular development, luteal function, and early embryo survival, thereby leading to reduced pregnancy rates [37,38].

In extensive plateau grazing systems, direct assessment of body weight or BCS is often impractical. Chest girth serves as a robust, field-friendly proxy for body condition, correlating strongly with both skeletal size and fat reserves in ruminants [39]. In this context, chest girth represents a simple, low-cost, and highly repeatable indicator of body condition. Previous studies have demonstrated that chest girth shows the strongest correlation with body weight among commonly used body measurements, and the inclusion of additional variables often provides limited improvement over models based solely on chest girth [38]; Furthermore, Xiong reported that image-based and morphometric approaches using simple body measurements, including chest girth, can accurately estimate body weight and BCS in adult beef cattle, thereby indirectly reflecting energy reserve status [40]. Collectively, these findings indicate that body measurements are important predictors of reproductive potential. Given the strong positive correlations between chest girth, body weight, and BCS in cattle, chest girth can serve as an effective parameter for predicting estrus and pregnancy outcomes [18,41]. Consistent with these observations, the present study found that female yaks with larger chest girth exhibited higher estrus and pregnancy rates. This finding underscores the importance of maintaining adequate body condition to improve reproductive efficiency in yaks and supports the use of chest girth as a practical and easily accessible indicator for estimating energy reserves and predicting reproductive performance.

Importantly, this study revealed that the CIDR-GPG protocol reduced the chest girth threshold required for successful estrus expression and pregnancy establishment. This indicates that even yaks with relatively moderate body condition can achieve favorable reproductive outcomes under this hormonal regimen. Such a reduction in the physiological threshold effectively broadens the population eligible for TAI and highlights the capacity of CIDR-GPG to compensate, at least partially, for suboptimal body condition through endocrine regulation. This finding represents a key practical advantage of the CIDR-GPG protocol in plateau yak production systems.

Several limitations should be acknowledged. Due to the harsh plateau environment and field conditions, complete body weight data could not be obtained, and chest girth was used as a proxy for body condition. Although chest girth correlates strongly with reproductive outcomes, it should be interpreted as a comprehensive proxy for the animal’s nutritional and metabolic status rather than a direct causal factor. In addition, several potential confounding factors inherent to field-based studies were not fully controlled and may have influenced the results. An a priori statistical power calculation was not conducted due to logistical constraints and limited animal availability. Post hoc power analysis yielded values of 0.65 for estrus rate and 0.72 for pregnancy rate. Future follow-up research should expand the sample size, establish a natural estrus control group, and conduct direct detection of physiological indicators and metabolic profiles.

## 5. Conclusions

In conclusion, this study suggests that the CIDR-GPG protocol is the most effective among the tested treatments for estrus synchronization in plateau yaks. Our findings highlight chest girth as a reliable and accessible indicator for reproductive management. Crucially, we revealed that the CIDR-GPG protocol lowered the body condition threshold for success, extending the benefits of TAI to a larger population. Implementing this protocol in combination with chest girth assessment may provide a practical approach to improve reproductive efficiency in yak herds.

## Figures and Tables

**Table 1:** Comparison of body measurements of yaks among the 3 estrus synchronization protocols.

Indicator	CIDR-GPPG		CIDR-GPG		GPPG	
	Mean ± SEM	CV/%	Mean ± SEM	CV/%	Mean ± SEM	CV/%
Body Height/cm	106.1 ± 1.0 ^a^	5.3	105.6 ± 1.0 ^a^	5.4	104.6 ± 1.0 ^a^	5.8
Body Straight Length/cm	111.4 ± 0.8 ^a^	4.3	110.9 ± 1.0 ^a^	6.3	110.1 ± 0.9 ^a^	4.8
Body Slope Length/cm	117.4 ± 1.0 ^a^	5.8	116.2 ± 1.0 ^a^	6.6	116.2 ± 1.0 ^a^	6.0
Head Length/cm	38.5 ± 0.4 ^a^	5.6	38.6 ± 0.8 ^a^	11.9	38.9 ± 0.2 ^a^	3.3
Sacral Height/cm	97.6 ± 0.7 ^a^	4.2	98.5 ± 1.0 ^a^	5.7	96.9 ± 0.8 ^a^	4.8
Cannon Girth/cm	14.2 ± 0.2 ^a^	9.2	14.6 ± 0.2 ^a^	7.1	14.3 ± 0.1 ^a^	5.3
Chest Girth/cm	144.6 ± 1.0 ^a^	5.9	144.8 ± 2.0 ^a^	7.1	142.6 ± 2.0 ^a^	6.8
Abdominal Girth/cm	162.0 ± 2.0 ^a^	7.8	165.0 ± 2.0 ^a^	8.9	163.2 ± 2.0 ^a^	7.6
Hip Width/cm	29.7 ± 0.5 ^a^	9.4	29.9 ± 0.5 ^a^	10.1	29.8 ± 0.4 ^a^	7.2
Pelvic Width/cm	11.1 ± 0.3 ^a^	15.4	11.1 ± 0.3 ^a^	15.9	11.4 ± 0.2 ^a^	8.9

CIDR-GPG: controlled internal drug release device combined with GnRH-PGF2α-GnRH synchronization protocol; CIDR-GPPG: controlled internal drug release device combined with GnRH-PGF2α-PGF2α-GnRH synchronization protocol; GPPG: GnRH-PGF2α-PGF2α-GnRH synchronization protocol. ^a^ Values with the same letters within a row do not significantly differ (*p* > 0.05). Values are presented as mean ± SEM.

**Table 2:** Chi-square analysis of estrus rates among the 3 estrus synchronization protocols.

Indicator	Overall	CIDR-GPPG	CIDR-GPG	GPPG
Estrus Rate (%)	52.5	50.0	65.7	40.6
Sample Size (Success/Total)	52/99	16/32	23/35	13/32
Chi-square Test Results	CIDR-GPPG vs. CIDR-GPG: χ^2^ = 1.701, df = 1, *p* = 0.193 CIDR-GPPG vs. GPPG: χ^2^ = 0.568, df = 1, *p* = 0.451 CIDR-GPG vs. GPPG: χ^2^ = 4.22, df = 1, *p* = 0.040			

**Table 3:** Chi-square analysis of pregnancy rates among the 3 estrus synchronization protocols.

Indicator	Overall	CIDR-GPPG	CIDR-GPG	GPPG
Pregnancy Rate (%)	38.5	37.5	52.2	15.4
Sample Size (Success/Total)	20/52	6/16	12/23	2/13
Chi-square Test Result	CIDR-GPPG vs. CIDR-GPG: χ^2^ = 0.818, df = 1, *p* = 0.366 CIDR-GPPG vs. GPPG: χ^2^ = 1.756, df = 1, *p* = 0.185 CIDR-GPG vs. GPPG: χ^2^ = 4.730, df = 1, *p* = 0.030			

**Table 4:** Comparison of body measurements between estrus and non-estrus female yaks.

Indicator	Estrus		Non-Estrus	
	Mean ± SEM	CV/%	Mean ± SEM	CV/%
Body Height/cm	108.3 ± 0.7 ^a^	4.4	102.3 ± 0.7 ^b^	5.0
Body Straight Length/cm	114.2 ± 0.6 ^a^	4.3	107.1 ± 0.7 ^b^	3.8
Body Slope Length/cm	120.7 ± 0.8 ^a^	4.9	112.0 ± 0.8 ^b^	4.8
Head Length/cm	39.1 ± 0.2 ^a^	10.0	38.2 ± 0.5 ^a^	4.0
Sacral Height/cm	100.3 ± 0.5 ^a^	4.3	94.8 ± 0.6 ^b^	3.8
Cannon Girth/cm	14.7 ± 0.1 ^a^	6.8	14.0 ± 0.1 ^b^	7.2
Chest Girth/cm	150.5 ± 1.0 ^a^	4.4	136.8 ± 0.9 ^b^	4.9
Abdominal Girth/cm	171.9 ± 1.0 ^a^	6.5	154.0 ± 2.0 ^b^	5.2
Hip Width/cm	30.1 ± 0.4 ^a^	8.2	29.5 ± 0.3 ^a^	9.7
Pelvic Width/cm	11.4 ± 0.2 ^a^	14.5	11.0 ± 0.2 ^b^	12.5

^a,b^ Values with the same letters within a row do not significantly differ (*p* > 0.05), whereas values with different letters within a row are significantly differ (*p* < 0.05). Values are presented as mean ± SEM.

**Table 5:** Classification performance of the chest girth–based logistic regression model for estrus prediction in female yaks.

Index	Accuracy	Precision	Recall	F1
Value	0.889	0.902	0.885	0.893

**Table 6:** Comparison of body measurements between pregnant and non-pregnant female yaks.

Indicator	Pregnant		Non-Pregnant	
	Mean ± SEM	CV/%	Mean ± SEM	CV/%
Body Height/cm	107.0 ± 0.8 ^a^	4.4	104.5 ± 1.0 ^a^	4.1
Body Straight Length/cm	117.8 ± 1.0 ^a^	5.6	109.3 ± 1.0 ^b^	5.3
Body Slope Length/cm	121.3 ± 1.0 ^a^	4.5	115.4 ± 1.0 ^b^	5.5
Head Length/cm	39.5 ± 0.8 ^a^	6.1	38.9 ± 0.5 ^a^	12.0
Sacral Height/cm	103.2 ± 1.0 ^a^	6.4	97.5 ± 1.0 ^b^	6.9
Cannon Girth/cm	14.8 ± 0.2 ^a^	8.1	14.6 ± 0.3 ^a^	6.0
Chest Girth /cm	155.4 ± 1.0 ^a^	5.1	138.7 ± 2.0 ^b^	5.8
Abdominal Girth/cm	177.7 ± 2.0 ^a^	5.0	160.1 ± 2.0 ^b^	6.3
Hip Width/cm	30.2 ± 0.4 ^a^	9.1	30.1 ± 0.6 ^a^	7.8
Pelvic Width/cm	11.2 ± 0.2 ^a^	18.9	11.6 ± 0.5 ^a^	11.2

^a,b^ Values with the same letters within a row do not significantly differ (*p* > 0.05), whereas values with different letters within a row are significantly differ (*p* < 0.05). Values are presented as mean ± SEM.

**Table 7:** Classification performance of the chest girth–based logistic regression model for pregnancy prediction in female yaks.

Index	Accuracy	Precision	Recall	F1
Value	0.904	0.857	0.900	0.878

**Table 8:** Classification performance of chest girth–based logistic regression models for estrus and pregnancy prediction in female yaks under the CIDR-GPG protocol.

Index	Accuracy	Precision	Recall	F1
Estrus	0.829	0.870	0.870	0.870
Pregnancy	0.826	0.833	0.833	0.833

## Data Availability

The original contributions presented in this study are included in the article. Further inquiries can be directed to the corresponding author.

## Supplementary Materials

The following supporting information can be downloaded at: https://www.mdpi.com/article/10.3390/ani16111582/s1, Table S1. Feed composition and nutrient levels of the supplementary diets for lactating and pregnant yaks. Table S2. Sperm quality parameters of frozen–thawed yak semen from three different batches.
