# Supplementary material for: The CIDR-GPG Protocol Improves Reproductive Efficiency in Yaks and Lowers the Body Condition Requirements for Success
Source: Animals (Basel). 2026 May 22;16(11):1582. doi: 10.3390/ani16111582 (PMC13256044; doi:10.3390/ani16111582)
Supplement: Supplementary file 1 [file animals-16-01582-s001.zip › animals-4251140-supplementary.pdf]

Supplementary Table S1. Feed composition and nutrient levels of the supplementary diets for lactating and pregnant yaks.

Lactating yak			Pregnant yak		
Concentrated feed formula		TMR diet	Concentrated feed formula		TMR diet
Premixed feed	Concentrated feed		Premixed feed	Concentrated feed	
Salt 20kg	Corn 500kg	Oatmeal 3kg	Salt 10kg	Corn 400kg	Oatmeal 3kg
Stone powder 30kg	Soybean meal 200kg	Alfalfa 2kg	Stone powder 20kg	Soybean meal 300kg	Alfalfa 1kg
Dicalcium phosphate 15kg	Highland barley powder 220kg	corn stalk 1kg	Dicalcium phosphate 10kg	Highland barley powder 245kg	corn stalk 2kg
Trace element 10kg		Concentrated feed 3.5kg	Trace element 10kg		Concentrated feed 2.5kg
Vitamin 5kg			Vitamin 5kg		

Values are expressed as kg per head per day. The diets consisted of concentrate and total mixed ration (TMR) components formulated for lactating and pregnant yaks.

Supplementary Table S2. Sperm quality parameters of frozen﹣thawed yak semen from three different batches.

	First batch：0607	Second batch：0705	Third batch：0804
Total vitality	51.80%[a]	62.15%[b]	74.15%[b]
vitality	21.88%[a]	16.90%[b]	22.60%[a]
bent tail sperm	14.25%[a]	18.87%[b]	18.32%[b]
coiled tail sperm	0.50%[a]	0.23%[b]	0.40%[a]
DMR	3.13%[a]	2.88%[a]	2.32%[b]
Distal droplet	16.98%[a]	22.17%[b]	21.20%[b]
Proximal drop	4.30%[a]	9.47%[b]	7.55%[b]
Normal percentage	83.55%[a]	80.65%[a]	79.67%[a]
Deformity rate	16.45%[a]	19.35%[a]	20.33%[a]
Density (millions /0.25 mL)	67.52[a]	80.16[b]	83.10[b]
Sperm number of forward movement (millions)	22.61[a]	26.61[a]	26.99[a]

Values are presented as mean percentages or concentrations per 0.25 mL semen straw.

[a-b]Values with the same letters within a row do not significantly differ ($P > 0.05$)，whereas values with different letters within a row are significantly differ ($P < 0.05$).
